# Removal of Photosystem I Subunit PsaE Leads to Photosynthetic Hydrogen Production in a Cyanobacterial PsaF‐HoxYH Fusion Strain

**DOI:** 10.1111/pce.70659

**Published:** 2026-06-09

**Authors:** Florian Paul, Nadine Strabel, Marko Boehm, Jens Appel, Kirstin Gutekunst

**Affiliations:** ^1^ Molecular Plant Physiology, Bioenergetics in Photoautotrophs University of Kassel Kassel Germany

## Abstract

Photosynthetic hydrogen (photoH_2_) production is a promising approach for the utilisation of solar energy. Fusion of a hydrogenase (H_2_ase) to photosystem I (PSI) provides a direct light‐driven route for photoH_2_ generation. We established the lateral transmembrane subunit PsaF of PSI as a new fusion partner for the NiFe‐hydrogenase (HoxYH) in the cyanobacterium *Synechocystis* sp. PCC 6803. Gradual reduction of PsaC expression in a PsaF‐HoxYH strain was established to access the most redox negative FeS‐cluster F_X_ of PSI, but this did not result in photoH_2_ production. In another approach PSI subunit PsaE was identified as a potential steric hindrance in the psaF‐hoxYH strain to electron transfer between the FeS‐cluster F_B_ cluster in PSI and HoxYH. Consistent with this, deletion of PsaE in psaF‐hoxYH led to successful photoH_2_ production. Our results identify PsaF as a viable and structurally favourable anchor providing a new strategy for the engineering of photobiological H_2_‐production systems.

## Summary statement

The lateral photosystem I (PSI) subunit PsaF was established as a new anchor for the fusion of the NiFe‐hydrogenase and potentially other electron accepting enzymes in the cyanobacterium *Synechocystis* sp. PCC 6803.

The direct conversion of solar energy into hydrogen offers a sustainable route to reduce fossil fuel dependency. Photosynthesis has always formed the basis of human energy supply: it not only produces carbohydrates that sustain metabolism but also gives rise to fossil carbon reserves over geological time. Redirecting photosynthetically excited electrons toward hydrogen generation allows solar energy to be harvested with high efficiency, avoiding less efficient carbon‐based intermediates.

Hydrogen production occurs naturally in some phototrophic microorganisms. Cyanobacteria employ NiFe‐hydrogenases (H_2_ases), while green algae contain FeFe‐H_2_ases, enabling both groups to release molecular hydrogen (Redding et al. 2022). This can take place under anaerobic, fermentative conditions in darkness, or as light‐driven photosynthetic hydrogen evolution (photoH_2_) when cells transition from dark to illuminated states.

Cyanobacterial photosystem I (PSI) is a multisubunit complex in the thylakoid membrane composed of transmembrane and stromal subunits (PsaA‐F, I, J, K, L, M, X). The FeS‐cluster F_X_ is located in the core of the PsaA/PsaB heterodimer at the interface of PsaC, while the FeS‐clusters F_A_, and F_B_ are localised on PsaC. Upon light absorption, an internal electron transfer in PSI from the special chlorophyll pair P700 via another chlorophyll pair A_0_, phylloquinone A_1_ and the FeS‐clusters F_X_, F_A_, and F_B_ causes a charge separation between P700 and F_B_ with a voltage difference of 1 V. Subsequently, F_B_ reduces soluble ferredoxins, which distribute electrons to various reactions, such as the Calvin‐Benson‐Bassham (CBB) cycle for CO_2_ fixation. Fusion of H_2_ases to PSIs instead diverts electrons to photoH_2_ production.

A successful production system for photoH_2_ uses the green algae *C. reinhardtii*, where the native FeFe‐H_2_ase (HydA) was fused to PSI subunit PsaC (Kanygin et al. 2020). For the in vivo fusion of the NiFe‐ H_2_ase (Hox) in the cyanobacterium *Synechocystis* sp. PCC 6803 (hereafter *Synechocystis*), the PSI subunits PsaC, PsaD or PsaE were used (Redding et al. 2022; Appel et al. 2020; Wang et al. 2023). The respective PSI‐H_2_ase fusions have been characterised in vivo and in vitro (Appel et al. 2020; Wang et al. 2023). While it was possible to insert the FeFe‐ H_2_ase into a loop of PsaC in a position close to the F_B_‐cluster, enabling high electron transfer rates (Kanygin et al. 2020), this is not possible for the NiFe‐H_2_ases due to a long distance (> 30 Å) between its N‐terminus and the FeS‐cluster in the HoxY subunit. Nevertheless, NiFe‐H_2_ases are valuable candidates for such fusions since they are—in contrast to FeFe‐H_2_ases—able to cope with low (McIntosh et al. 2011) or even ambient oxygen concentrations (Lenz et al. 2018; Lupacchini et al. 2025).

To divert electrons from PSI for light‐driven H_2_ production, the cofactor with the most negative redox potential would be best in terms of energetic driving force. However, the probability of electron transfer to the H_2_ase also depends on the lifetime of the electron on the respective cofactor before charge recombination with P700^+^ occurs (Gorka and Golbeck 2020; Gorka et al. 2015). All in vivo approaches with PSI‐H_2_ase fusion mutants to date utilised the F_B_ cluster of PSI as an electron donor for H_2_ production (Redding et al. 2022; Kanygin et al. 2020; Appel et al. 2020; Wang et al. 2023). No attempts have been made to use the more negative F_X_ cluster as an electron donor for a H_2_ase so far.

In this study, we present the successful in vivo fusion of the NiFe‐H_2_ase subunit HoxYH of *Synechocystis* to a truncated version of the PSI subunit PsaF. Two PsaF‐H_2_ase variants were tested. One variant targeted the more redox‐negative F_X_ cluster (−620 to −700 mV), but without success, while the other variant utilised the conventional F_B_ cluster (−530 to −560 mV), which led to photoH_2_ production. This establishes the PSI subunit PsaF as a new anchor for H_2_ases.

Previously, the PSI‐H_2_ase fusion mutant *psaD‐hoxYH* appeared as a promising mutant to further optimise photoH_2_ production (Appel et al. 2020). However, this mutant turned out to be unstable in its H_2_ase activity, which became noticeable by a decrease of photoH_2_ production over time. While H_2_ase activity in methyl viologen assays, which quantify active H_2_ases by providing artificial electron donors, persisted at near‐WT levels for some clones, others lost all activity (Figure S1a). For clones with remaining H_2_ase activity in MV‐assays, only a small fraction of previous photoH_2_ production remained (Figure S1b). This result is a major concern for the long‐term stability of PSI‐H_2_ase mutants for biotechnological approaches. Therefore, the transmembrane subunit PsaF, which is located to the side of the PsaA/PsaB heterodimer, was explored as a novel fusion partner (Figure S2).

Since the C‐terminus of PsaF points towards the central PsaC‐subunit, which contains the F_B_ cluster, it provides a favourable starting point for fusing the H_2_ases. For the assembly of the 4Fe4S cluster of HoxY into proximity to either the F_X_ or the F_B_, we fused HoxY to Val154 of PsaF, deleting the last 11 C‐terminal amino acids of PsaF and replacing it with a 12 amino acid flexible linker ([GGS] ×4), which was designed with considerations from Alphafold modelling (Figure S2). In this new configuration, the PsaE subunit was identified in silico as a potential steric hindrance to access the F_B_ cluster. To test this, we constructed two background strains for the integration of the psaF‐hoxYH fusions: Δ*hox* and Δ*psaE*Δ*hox*. For clarity, the resulting mutants will be called psaF‐hoxYH (in the Δ*hox* background strain) and psaF‐hoxYH ΔpsaE (in the Δ*psaE*Δ*hox* background strain). Based on the Alphafold modelling (Figure S2), our assumption was that photoH_2_ production would be higher in psaF‐hoxYH ΔpsaE in contrast to psaF‐hoxYH, due to an improved accessibility to the F_B_ cluster of PsaC.

To access the F_X_ as a potential electron donor for the H_2_ase, the three stromal subunits PsaC, PsaD and PsaE of PSI have to be removed. It was previously shown that in the absence of PsaC, none of the three stromal subunits assembles to the PsaA/PsaB core (Yu et al. 1995). Since a deletion strain of *psaC* is not viable under photoautotrophic conditions, we decided to introduce the rhamnose‐inducible promotor P_rha_ in the PsaC‐locus to create the mutant PsaF‐hoxYH ΔpsaE P_rha_‐PsaC. Thereby, this mutant could be switched between a state with expressed PsaC on rhamnose for photoautotrophic growth, and a low‐expression state without rhamnose to expose the F_X_‐cluster for photoH_2_ production. As an alternative to a strict switch between the two states, a mixed culture of cells containing photosystems in both states could also be potentially effective.

The final 13 amino acids of the PsaC C‐terminus (sequence: LGAETTRSMGLAY, henceforth referred to as PsaC‐tag) were attached to the C‐terminus of HoxY with a five‐amino acid flexible linker sequence (KESGS). This C‐terminus was previously determined as responsible for the binding of PsaC to PsaB (Antonkine and Golbeck 2006). The rationale behind this design was to anchor the fused hydrogenase N‐terminally to PsaF and to pull it C‐terminally via the PsaC‐tag into proximity of the F_X_‐cluster.

**Figure 1:**
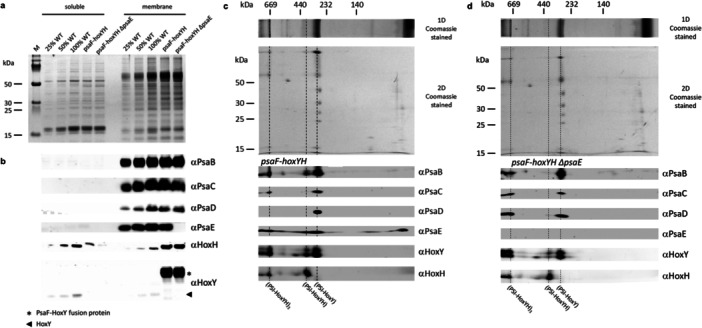
Protein analysis in WT, *psaF‐hoxYH*‐mutant and *psaF‐hoxYH ΔpsaE* mutant via immunoblot after 1D‐ and 2D‐PAGE. (a) Coomassie‐stained and unstained 5%–12% (w/v) polyacrylamide first dimension blue native polyacrylamide gel electrophoresis (1D BN–PAGE) gel lanes of the crude membrane fraction of ΔpsaE psaF‐hoxYH. (b) 1D‐ immunoblot analysis of soluble and membrane fractions shows assembly of psaF‐hoxYH fusion for psaF‐hoxYH and psaF‐hoxYH ΔpsaE mutants, as a shift in band size of HoxY (indicated by *) compared to the WT (indicated by ◄) and deletion psaE in psaF‐hoxYH ΔpsaE. (c, d) Coomassie stains of 1D SDS PAGE for psaF‐hoxYH (in b) and psaF‐hoxYH ΔpsaE (in c) in the upper lane, and Coomassie and immunoblot analysis of 2D SDS‐PAGE blots generated from a 1D BN‐PAGE lane below. In both samples, PSI monomers and trimers contain the fusion of PsaF–HoxYH as indicated.

Verification of the described mutants was carried out by immunoblotting. In accordance with expectations, the level of PsaB, PsaC and PsaD expression remained unchanged for both psaF‐hoxYH and psaF‐hoxYH ΔpsaE when compared to the WT (Figure 1a). Likewise, PsaB and PsaD expression remained unchanged for psaF‐hoxYH ΔpsaE P_rha_‐psaC (Figure S4a,b). Deletion of the *psaE* gene successfully removed PsaE in psaF‐hoxYH ΔpsaE (Figure 1a,c). HoxY was successfully attached to PsaF in all three mutants, as visible by its size shift in comparison to HoxY in the WT and its localisation to the membrane fractions (Figures 1 and S4c). Furthermore, PsaC expression could be modified by different rhamnose concentrations in psaF‐hoxYH ΔpsaE P_rha_‐psaC (Figure S4a,b).

**Figure 2:**
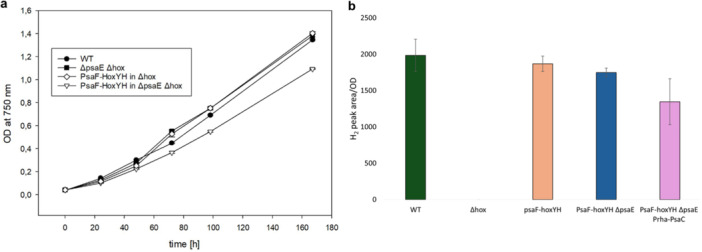
Growth and hydrogenase activity in WT, ΔpsaE Δhox, psaF‐hoxYH and psaF‐hoxYH ΔpsaE strains. (a) Photoautotrophic growth of WT, Δhox ΔpsaE, psaF‐hoxYH and psaF‐hoxYH ΔpsaE strains in continuous light at 50 µE m^−2^ s^−1^ measured by optical density at 750 nm. Mean values and standard deviations are shown (*n* = 3 for each of the four strains). (b) Hydrogenase assay in the presence of dithionite (DT) and reduced methyl viologen (MV) confirms expression of active hydrogenase for WT, psaF‐hoxYH, psaF‐hoxYH ΔpsaE and PsaF‐hoxYH ΔpsaE P_rha_‐PsaC (with 10 mM rhamnose). Measurements were taken with *n* = 3 for each sample. The H_2_ peak area per sample was determined from a 100 µl headspace sample of a 4 ml vial with a gas chromatograph (GC) after incubation.

To test the general fitness of the *psaF‐H*_*2*_ *ase* fusion strains, photoautotrophic growth was monitored. The mutants *ΔpsaEΔhox* and psaF‐hoxYH show identical photoautotrophic growth to the WT, while psaF‐hoxYH ΔpsaE was slightly impaired (Figure 2a). This provides a significant improvement compared to the *ΔpsaDΔhox* strain, which is impaired in photoautotrophic growth (Appel et al. 2020).

Functional H_2_ases were quantified by gas chromatography in MV‐assays that provide artificial electron donors to the enzymes. These assays confirmed the successful expression of functional H_2_ases in all three PsaF‐H_2_ase fusion strains (Figure 2b). A similar H_2_ peak area per OD_750_ was detected for psaF‐hoxYH at 94% and psaF‐hoxYH ΔpsaE at 88% of the WT value. Under cultivation with 10 mM of rhamnose, the mutant PsaF‐hoxYH ΔpsaE P_rha_‐PsaC produced 67% as much H_2_ as the WT. The maximum rates of H_2_ production were also quantified, applying the MV assay to samples measured with a Clark‐type electrode (Figure S3). WT and psaF‐hoxYH performed similarly with a rate of 10.3 and 9.5 µmol*min^−1^ OD_750_^−1^, respectively. At a slightly higher rate of 12.84 µmol*min^−1^ OD_750_^−1^, psaF‐hoxYH ΔpsaE performed similarly to the psaD‐hoxYH strain at 12 µmol*min^−1^ OD_750_^−1^ (Appel et al. 2020), suggesting a similar amount of assembled H_2_ase molecules per OD_750_.

**Figure 3:**
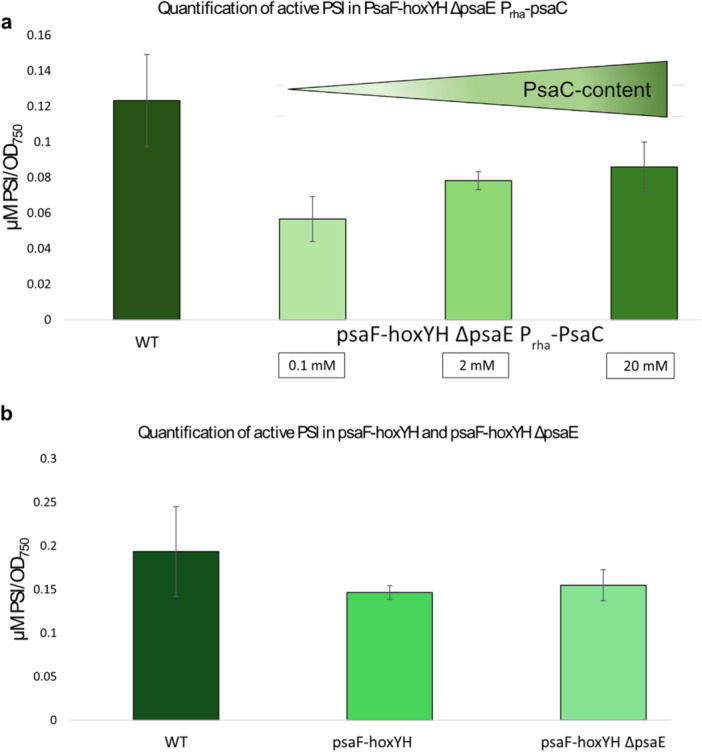
Active PSI quantified by the P700^+^ signal in Nir‐Max measurements with the DUAL‐KLAS‐NIR. (a) Active PSI in PsaF‐hoxYH ΔpsaE P_rha_‐PsaC cultures with varying rhamnose concentrations to downregulate the expression of PSI subunit PsaC. (b) Active PSI in WT, psaF‐hoxYH and psaF‐hoxYH ΔpsaE. Mean values and standard deviations (*n* = 3 for each strain) are shown.

PsaF‐hoxYH ΔpsaE P_rha_‐PsaC mutants were grown with different rhamnose concentrations to implement PsaC repression at low rhamnose concentrations for exposure of the F_X_ cluster. PSI activity was tested in relation to rhamnose concentrations (Figure 3a). The lowest induction level (0.1 mM rhamnose) of PsaC resulted in a decrease to about 46% active PSI (Figure 3a). As expected, the percentage gradually increased by providing more rhamnose‐induced PsaC‐expression to the cells, with 63.5% at 2 mM and 70% at 20 mM rhamnose, thus confirming successful regulation of the amount of PsaC subunits. For comparison, PSI activity was also determined in psaF‐hoxYH to 76% and in psaF‐hoxYH *ΔpsaE* to 80% total activity compared to WT (Figure 3b). The inducible expression is in a good range since it was shown that 25% of the wild‐type level of PSI is sufficient for photoautotrophic growth (Moore and Vermaas 2024).

The in vivo photoH_2_ production in PsaF‐H_2_ase mutants was assessed with Clark‐type H_2_ sensors under anaerobic conditions in the light. We chose to compare the strains in terms of their highest photoH_2_ production rates when they are shifted from dark anaerobiosis to light. Due to the transient acceptor side limitation of PSI under these conditions, there is no competition with ferredoxin and production rates should mirror the electron transfer rates inside the fusion complexes. These rates decreased after about 200 s, which might occur due to the activation of the CBB cycle and subsequent competition for electrons at the level of ferredoxin in these mutants.

**Figure 4:**
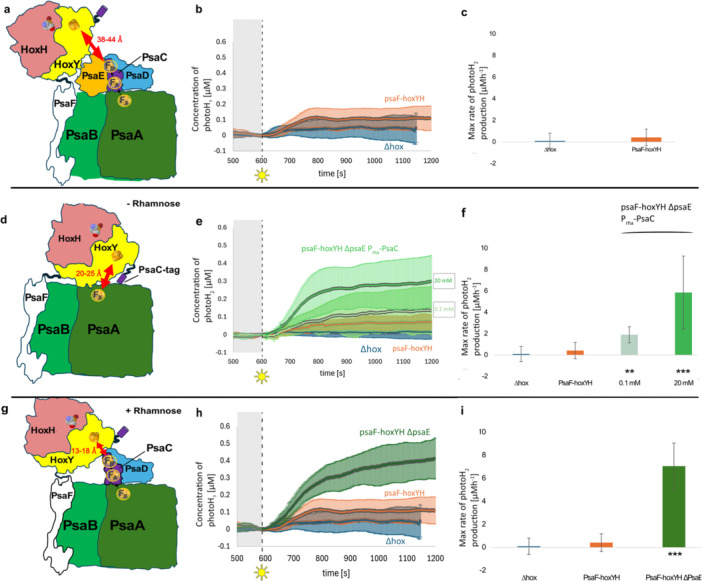
Schematic overview of PsaF‐hoxYH fusion*s* and photoH_2_ production upon illumination under anaerobic conditions. Anaerobic conditions were achieved through the addition of glucose, glucose oxidase and catalase. Actinic light at saturating levels was turned on after 600 s of darkness (indicated by the dashed line). (a) Schematic overview of the PSI‐fusion assembly in psaF‐hoxYH. Estimated distance between 4Fe4S clusters in HoxY and the F_B_ in PsaC (based on the cluster distance in Alphafold3 models) is highlighted in red. (b) PhotoH_2_ in *Δhox* and psaF‐hoxYH. The solid lines are the average of *n* = 9 measurements, and the surrounding lighter colour indicates the standard deviation. (c) Maximum rates of H_2_ production in (b). (d) Schematic overview of the PSI‐fusion assembly in psaF‐hoxYH ΔpsaE P_rha_‐PsaC under low rhamnose induction, thus lacking the subunits PsaC, PsaD and PsaE. Estimated distance between 4Fe4S clusters in HoxY and the F_X_ is highlighted in red. (e) PhotoH_2_ in *Δhox*, psaF‐hoxYH and psaF‐hoxYH ΔpsaE P_rha_‐PsaC under high (20 mM) and low (0.1 mM) rhamnose induction. (f) Maximum rates of photoH_2_ production in (e). ** indicates *p* < 0.05, *** indicates *p* < 0.01. (g) Schematic overview of the PSI‐fusion assembly in psaF‐hoxYH ΔpsaE and psaF‐hoxYH ΔpsaE P_rha_‐PsaC under high rhamnose induction. Estimated distance between 4Fe4S clusters in HoxY and the F_B_ in PsaC is highlighted in red. (h) PhotoH_2_ in *Δhox*, psaF‐hoxYH and psaF‐hoxYH ΔpsaE. (i) Maximum rates of H_2_ production in (h). The purple residues attached to HoxY, referred to as “PsaC‐tag”, were added as an attempt to pull the H_2_ase closer by binding to the surface of PsaA.

Low rates of photoH_2_ of about 0.43 µM h^−1^ were determined for the control strain psaF‐hoxYH (Figure 4). This was expected as the presence of PsaE sterically hinders electron transfer between the F_B_ cluster of PsaC and the FeS cluster in HoxY (Figure S2a).

In contrast to this, both mutants psaF‐hoxYH ΔpsaE P_rha_‐PsaC and psaF‐HoxYH ΔpsaE lack the potentially hindering PsaE subunit. These mutants were tested under various conditions, targeting either the F_X_ or F_B_ cluster as an electron donor.

To expose F_X_ as a potential electron donor, psaF‐hoxYH ΔpsaE P_rha_‐PsaC was incubated with 0.1 mM rhamnose to suppress PsaC (Figure 4d), which also prevents the attachment of PsaE and PsaD to PSI (Figure 4d) (Yu et al. 1995). Under these conditions, slightly higher rates of 1.9 µM h^−1^ photoH_2_ were detected in this strain (Figure 4e).

To target F_B_ as an electron donor, psaF‐hoxYH ΔpsaE P_rha_‐PsaC was cultivated in the presence of 20 mM rhamnose. As PsaC is expressed under these conditions (Figure S4a), psaF‐hoxYH ΔpsaE P_rha_‐PsaC should correspond to the mutant psaF‐hoxYH ΔpsaE in terms of photosystem structure and PSI‐H_2_ase fusion (Figure 4g). Accordingly, both mutants showed similar rates of 5.9 and 7 µM h^−1^ photoH_2,_ which are 14 and 16 times above the rates that were reached in the psaF‐hoxYH mutant in the presence of PsaE.

The subunits PsaC, PsaD and PsaE sit atop the PSI core, where they form the ferredoxin binding site (Kubota‐Kawai et al. 2018; Li et al. 2022) (Figure S6). While a recent study found an incomplete F_X_ in the absence of PsaC in in‐vitro PSI preparations (Charras Ferroussier et al. 2025), functional F_X_ assembly was observed in vivo in *Synechocystis* Δ*psaC‐*strains (Gong et al. 2003, 2009).

With its more negative redox potential of −650 mV, the F_X_ cluster offers greater driving force for electron transfer than the F_B_ cluster (−550 mV) (Kanda and Ishikita 2023). However, this comes with the downside of a faster recombination time of 1–5 ms. An even faster recombination time of 750 ± 250 µs was reported for F_X_‐exposed PSI in the absence of PsaC, PsaD and PsaE (Kruip et al. 1993) and might be attributed to two more net negative charges at the surface of PsaAB in the absence of PsaC (Gong et al. 2003). PhotoH_2_ production aiming at utilising the F_X_‐cluster is therefore especially challenging and requires optimal positioning of the H_2_ase with its FeS Cluster in a distance ≤ 14 Å to F_X_ (Page et al. 1999). The F_B_ retains electrons longer with a recombination time around 65 ms (Gorka et al. 2015), improving the prospect of electron transfer in suboptimal conditions (e.g. at cluster distances exceeding 14 Å).

Taking these considerations into account, electron flow between the F_X_ cluster and the FeS cluster of the H_2_ase appears unlikely in our fusion strains. Repression of PsaC (Figure 3), which exposes the F_X_ cluster, displayed lower photoH_2_ yields in comparison to mutants with higher PsaC containing the F_B_ cluster (Figure 4). Therefore, it is highly likely that the low photoH_2_ production of mutants with suppressed PsaC is due to remaining PsaC levels and electron transfer from F_B_ to the H_2_ase.

A major benefit of the F_B_ approach is the relative ease of cultivation of the strains (Figure 2a). F_X_‐exposed mutants face a detrimental impact on their photoautotrophic growth and are likely to experience defects in PSI content and activity. This could only be overcome by an inducible expression of PsaC as used in this study.

In conclusion, although the gradual reduction in PsaC expression to expose F_X_ in PSI did not lead to photoH_2_ production, this approach provides a platform for further optimisation steps. We furthermore succeeded in generating *psaF‐hoxYHΔpsaE* mutants that utilise the F_B_ cluster for photoH_2_ production and grow better under photoautotrophic conditions than the previously characterised *psaD‐hoxYH* fusion mutants. Their photoH_2_ production is comparable to that of *psaD‐hoxYH*. As an improvement to PsaE‐H_2_ase fusions, the attachment of the H_2_ase to the lateral PSI subunit PsaF offers greater flexibility for further fusion designs. We emphasise the promising assembly state of the psaF‐hoxYH ΔpsaE mutant at an estimated distance of 14–18 Å between F_B_ and the FeS‐clusters in HoxY, while maintaining functional hydrogenase activity. Due to the easy accessibility of the PsaF C‐terminus, various design options, such as trimming and addition of PSI‐homologue binding tags, become possible. This makes the PsaF‐fusion system an interesting candidate for other PSI‐coupling applications beyond hydrogen production, particularly those that require efficient electron transfer to catalytic partners. The observed photoH_2_ production, therefore, serves as evidence of functional electron transfer in this system and should be regarded as a proof of concept for a PSI‐based electron delivery platform. The proper design of PSI‐enzyme fusions for ideal FeS‐cluster proximity remains the most crucial step toward optimising light‐driven reduction of target compounds.

## 1 Experimental

### 1.1 Bioinformatics

Protein structure predictions were done using the publicly available Alphafold3 server at alphafoldserver.com. Default settings were used to generate predictions. Each query resulted in five predicted models, all of which were evaluated for each query. A representative model of their assembly was chosen for visualisation in Figures S2 and S5. The distance between FeS clusters of PSI and H_2_ase was measured using PyMOL by measuring the clusters within each model individually to annotate the variation across all five models.

### 1.2 Construction of Plasmids

All the primers that were used are listed in Table S1. The PCR products of the *hox* operon, *psaC and psaF* were generated along with those of either erythromycin or spectinomycin resistance cassettes. Two vectors were constructed: the psaF‐hoxYH expression vector (construct I) and the pP_Rha_‐PsaC vector to facilitate inducible regulation of PsaC (construct II). Their respective PCR products were joined in a single reaction into pBluescript or pMQ80 by Gibson assembly (Gibson et al. 2009).

To generate a fusion construct for PsaF‐hoxYH, four PCR products were generated: the *psaF*‐operon until the desired fusion site, the hoxY gene with 5' and 3' extensions encoding linker sequences and a C‐terminal PsaC‐tag, and the hoxH gene with a 5' extension encoding a twin‐strep‐tag were amplified and assembled into pMQ80 (Shanks et al. 2006), which was cut with EcoRI and NaeI in Gibson assembly. This is construct I. The introduction of the rhamnose promotor into the *psaC* operon was prepared by amplification of three PCR products: the upstream region of *psaC*, a cassette from pSHDY (Taton et al. 2014) consisting of P_rha_ with its transcription activator *RhaS* and a spectinomycin resistance gene, as well as the *psaC* gene with its downstream region. This resulted in pΔPSAC:P_Rha_‐PsaC, referred to as construct II.

Fragments had overlaps of 20 base pairs between them. A total of 50 ng of plasmid was combined in a 1:3 molar ratio with the different fragments. Chemically competent *Escherichia coli* DH5α was transformed with the resulting DNA and selected on LB‐plates containing the respective antibiotic. Plasmids were purified from the resulting colonies by growing 5 ml LB liquid cultures with the selective antibiotic overnight and then applying the NucleoSpin Plasmid Kit for plasmid DNA from Macherey‐Nagel according to the manufacturer's instructions. All of the obtained constructs were validated by sequencing.

### 1.3 Generation and Growth of Strains

Wild‐type cells of *Synechocystis* sp. PCC 6803 were transformed according to (Williams 1988) with pΔHOX and pΔPSAE to sequentially delete all the *hox* genes and *psaE*. The *Δhox ΔPsaE* strain was constructed as described earlier (Wang et al. 2023). Wild‐type, *Δhox* and *Δhox ΔPsaE* cells were grown at 50 µE m^–2^ s^–1^ on BG‐11 agar plates and in air‐bubbled liquid cultures. The *ΔhoxΔpsaE* strain was grown at 50 µE m^–2^ s^–1^ in air‐bubbled liquid cultures and transformed with construct I before plating them on BG‐11 with 25 µg ml^−1^ erythromycin. After segregation of the *ΔhoxΔpsaE psaF‐hoxYH* strain, cells that were additionally transformed with construct II were grown at 50 µE m^–2^ s^–1^ on BG‐11 plates containing 10 mM rhamnose and 20 µg ml^−1^ spectinomycin for segregation. The *psaF‐hoxYH* strain was generated by transforming construct I directly into the *Δhox* strain and selecting on BG‐11 plates with 25 µg ml^−1^ erythromycin.

For each transformation, cells were grown in shaking cultures (100 rpm) for 24 h at 50 µE m^–2^ s^–1^ before plating them on the selective BG‐11 plates. Plates were kept at 50 µE m^–2^ s^–1^ for several weeks before colonies appeared. Segregation of all resulting strains was checked by PCR reaction of genomic DNA. In the cases of *Δhox* and the fusion strains, successful transformation was also checked by measuring their hydrogenase activity.

### 1.4 Hydrogen Measurements

Hydrogenase activity was measured in whole cells in the presence of 10 mM dithionite and 5 mM MV with a hydrogen electrode as described previously (Gutekunst et al. 2018). The cells were grown under photoautotrophic conditions until they reached an OD_750_ of 1. They were then removed without further treatment for hydrogenase activity measurements. The resulting hydrogen in this MV assay was then quantified by analysis with a gas chromatograph (GC; Nexis‐GC 2030, Shimadzu) for activity screening, or by measuring with a Clark‐type electrode sensor to determine the production rate. For GC measurements, 3 ml of sample with an OD = 4 equivalent of cells were measured in 4 ml vials by incubating the samples in an end‐over‐end rotator for 20 min and then using 100 µl of the 1 ml headspace per sample in GC analysis. Fermentative and short‐term hydrogen turnovers under illumination were measured with the MicroRespiration System (Unisense). Cells grown at 50 µE m^–2^ s^–1^ with constant air bubbling were collected by centrifuging for 5 min at 8000 rcf and resuspended in fresh BG‐11_0_. The chlorophyll content was determined (Gutekunst et al. 2018) and adjusted to 12.5 µg ml^–1^. Cell suspensions of 1.6 ml (containing a total of 20 µg chlorophyll) were placed in 2 ml glass cuvettes and glucose, glucose oxidase and catalase were added at final concentrations of 10 mM, 16 U ml^–1^ and 20 U ml^–1^, respectively. Measurements were started after inserting a hydrogen electrode into each sample. All photoH_2_ samples were measured under light‐saturated conditions at an illumination of 800 µmol m^−2^ s^−1^. The hydrogen concentrations were continuously monitored in the aqueous phase of cyanobacterial cultures.

### 1.5 Protein Extract Preparation, Polyacrylamide Gel Electrophoresis and Immunoblotting

Cells were collected by centrifuging and resuspended in ACA buffer (750 mM ε‐aminocaproic acid, 50 mM BisTris/HCl, pH 7.0, 0.5 mM EDTA). Cells were broken by vortexing with glass beads (0.17–0.18 µm diameter) for 2 min at full speed at 4°C. Glass beads and cell debris were pelleted by centrifuging at 3000 × *g* for 10 min. Some whole cell extract (WCE) was centrifuged again at 20,000 × *g* for 20 min to separate crude membranes from soluble protein. The supernatant was kept as the soluble extract, and the crude membrane fraction (CMF) was resuspended in ACA buffer. Chlorophyll concentrations were determined for the WCE and the crude membrane samples. In the 1D SDS − PAGE immunoblot analysis, an amount corresponding to 10 µg total protein was loaded per lane. These samples were solubilised at room temperature for 1 h in 1x Laemmli sample buffer and separated on 12.5% (w/v) polyacrylamide BisTris gels using MES running buffer (250 mM MES, 250 mM Tris, 5 mM EDTA, 0.5% (w/v) SDS). For one 1D BN–PAGE gel lane, a CMF sample containing 2 µg chlorophyll was resuspended in 15 µl ACA buffer, and membranes were solubilised for 10 min at 4°C by the addition of 1.66 µl 10% (w/v) β‐dodecyl‐d‐maltoside. Insoluble material was pelleted by centrifugation at maximum speed for 10 min at 4°C in a microfuge. Subsequently, 1.6 µl Coomassie loading solution (750 mM ε‐aminocaproic acid, 5% (w/v) Coomassie‐G) was added and 18.2 µl was loaded per lane on 5%–12% (w/v) polyacrylamide BN PAGE first‐dimension gradient gel. For the 2D gels, the protein complexes separated in the first‐dimension gel strips were denatured for 1 h in solubilisation buffer (66 mM Na_2_CO_3_, 2% (w/v) SDS, 2% (v/v) β‐mercaptoethanol, 4 M urea) prior to being layered on 12.5% (w/v) polyacrylamide 6 M urea 2D SDS polyacrylamide gels. All resultant gels were either Coomassie‐stained or electroblotted onto a nitrocellulose membrane. For immunoblotting, 5% (w/v) milk powder in 1x PBS‐T was used as a blocking solution, and all washing steps were performed with 1x PBS‐T. The immunoblot analyses were performed using specific primary antibodies and horseradish peroxidase‐conjugated secondary antibodies (anti‐rabbit, GE Healthcare). Polyclonal, affinity‐purified primary HoxH and HoxY antibodies from rabbit raised against full‐length *E. coli* overexpressed protein were used (Eckert et al. 2012). The PsaB, PsaC and PsaD antibodies were purchased from Agrisera (Umeå) (Wittenberg et al. 2017).

### 1.6 PSI Activity Measurements

Total amount of active PSI was measured with a Dual‐KLAS/NIR (Klughammer and Schreiber 2016). Cell density was adjusted to 20 µg ml^−1^ chlorophyll, the samples were placed in a 1 × 1 cm^2^ cuvette and measured with the NirMax programme. During this programme, the cells are illuminated with the highest available far‐red light intensity for 10 s. Before light off, a 200 ms multiple turnover pulse (MT, of > 10,000 µE m^−2^ s^−1^) is given. Due to the far‐red light, which preferentially excites PSI, a large fraction of the P700 is already pre‐oxidised and becomes fully oxidised during the MT. The difference between the maximum signal and the signal after light off corresponds to the total amount of PSI in the sample. Using the conversion factor as determined in (Theune et al. 2021), the total concentration of PSI in µM can be calculated.

## Conflicts of Interest

Authors declare that the University of Kassel is holder of the patent PCT/EP2020/084252 “Genetically modified phototrophic cells for in‐vivo production of hydrogen”, with Jens Appel and Kirstin Gutekunst being the inventors.

## Supporting information


**Supporting File 1:** pce70659‐sup‐0001‐Supporting_Information_Postreview.docx.

**Supporting File 2:** pce70659‐sup‐0002‐Figure_S1.

**Supporting File 3:** pce70659‐sup‐0003‐Figure_S2.

**Supporting File 4:** pce70659‐sup‐0004‐Figure_S3.

**Supporting File 5:** pce70659‐sup‐0005‐Figure_S4.

**Supporting File 6:** pce70659‐sup‐0006‐Figure_S5.

**Supporting File 7:** pce70659‐sup‐0007‐Figure_S6.

**Supporting File 8:** pce70659‐sup‐0008‐Table_S1.

## Data Availability

The data that support the findings of this study are available in the supporting material of this article.
