# Supplementary material for: Removal of Photosystem I Subunit PsaE Leads to Photosynthetic Hydrogen Production in a Cyanobacterial PsaF‐HoxYH Fusion Strain
Source: Plant Cell Environ. 2026 Jun 9;49(9):6850–8. doi: 10.1111/pce.70659 (PMC13436535; doi:10.1111/pce.70659)
Supplement: Supplementary file 1 [file PCE-49-6850-s004.docx]

# Supporting information

Removal of Photosystem I Subunit PsaE Leads to Photosynthetic Hydrogen Production in a Cyanobacterial PsaF-HoxYH Fusion Strain

Florian Paul, Nadine Strabel, Marko Boehm, Jens Appel, Kirstin Gutekunst*

Molecular Plant Physiology, Bioenergetics in Photoautotrophs, University Kassel, Germany

*Corresponding Author: Kirstin Gutekunst [kirstin.gutekunst@uni-kassel.de](mailto:kirstin.gutekunst@uni-kassel.de)

Figure S1: Hydrogenase activity, photoH_2_ production and photoH_2_ production rate of *Δhox* and *psaD-hoxYH* mutants. **a.** Qualitative hydrogenase-assay in the presence of dithionite (DT) reduced methyl viologen (MV) reveals hydrogenase activity for some clones of psaD-hoxYH (D4, D6, D8, D10). The total amount of hydrogen produced is represented by the peak area determined using a gas-chromatograph (GC), which is displayed as a bar for each sample (n=1). Absolute values for the peak table are listed beneath each bar. **b. & c.** PhotoH_2_ production of the revived psaD-hoxYH mutant (b) versus data obtained in 2019 (in (c) as psaD-hoxYH 2019). One replicate was measured for Δhox, n=4 measurements of one clone (D10) were averaged for the psaD-hoxYH 2024 graph (b), while n=1 measurements of three separate clones are shown for the psaD-hoxYH 2019 data. The light source (emitting at 635 nm) was turned on at 600 s in (b) and at 400 s in (c), as indicated by the dashed lines. Each measurement was normalized to contain 20 µg of chlorophyll. **d**. Maximum rate of photoH_2_ production in (b&c). Data presented in orange in (b&d) were obtained by measuring a cryostock of the mutant psaD-hoxYH that was originally created in 2021 and revived in 2024. Maximum rates and standard deviation were determined through averaging the n=4 rates in (b) and the n=3 rates in (c).

Figure S2: Alphafold3 models for the designated PsaF-fusion approaches. **a.** psaF-hoxYH mutant with native PSI-core. The subunit PsaE is blocking access to the F_B_ as electron donor site. **b.** psaF-hoxYH ΔpsaE mutant with the accessible F_B_ as electron donor site**.** **c.** psaF-hoxYH ΔpsaE P_rha_-PsaC mutant at low rhamnose induction, resulting in PsaC-depletion and the exposed F_X_ as electron donor site. Present iron-sulfur clusters are depicted as orange/yellow spheres for each assembly.

Figure S3: Quantification of maximum H_2_ production rate in MV-assay. Hydrogenase assay in presence of dithionite (DT) and reduced methyl viologen (MV) confirms expression of active hydrogenase for WT and psaF-hoxYH Δhox (n=3) and psaF-hoxYH ΔpsaE Δhox (n=9). Measurements were taken from independent samples normalized to an OD_750_/ml=1 and with three technical replicates per biological replicate. The box plot shows the median as a solid line, the mean as dashed line and the upper and lower quartiles.

Figure S4: Immunoblot analysis of PSI-subunits in WT and PsaF-hoxYH ΔpsaE P_rha_-PsaC **a.** Coomassie-stain of WT and *psaF-hoxYH ΔpsaE P_rha_-PsaC* at varying induction stages. 2.5 µg of chlorophyll were loaded to each lane. **b.** Immunoblot analysis of PSI subunits PsaB, PsaC and PsaD in WT and *psaF-hoxYH ΔpsaE P_rha_-PsaC*. Four different rhamnose-induction states of the mutant were obtained by harvesting samples after 24 h or 72 h of cultivation in 0.1 mM or 10 mM rhamnose respectively. In the mutant samples, a significant decrease in PsaC expression is observed at both time points at low induction (0.1 mM) compared to WT. Expression of the genetically unmodified PsaB and PsaD subunits appeared to be stable. **c.** Immunoblot analysis of hydrogenase subunit HoxY in soluble and membrane fractions of identical samples to (a) and (b). A clear shift in band size of HoxY in the mutant (indicated by *) compared to the WT (indicated by ◄) was observed, thus confirming successful expression of the PsaF-HoxY fusion protein.

Figure S5: Alphafold3 models of PsaC-binding compared to experimental PSI structure **a.** Alphafold3 prediction of PsaA and PsaB together with the final C-terminal 13 amino acids of PsaC. **b.** Alphafold3 prediction of PsaA and PsaB together with the final C-terminal 20 amino acids of PsaC. **c.** Experimentally determined structure of PSI from Synechocystis (PDB: 6HQB). Subunits other than PsaA, PsaB and the final C-terminal 30 amino acids of PsaC are excluded from view for clarity. All three complexes are arranged in a top-down view with PsaA in green, PsaB in blue and PsaC in magenta. The AF3 prediction in (b) closely aligns to (c) regarding the assembly of PsaC. The shorter 13 amino acid residue of PsaC, as used with psaF-hoxYH fusions, is incorrectly assembled in the AF3 prediction in (a).

The intended binding was not observed in Alphafold models, presumably because the 13 amino acid sequence is too short to properly simulate folding and binding behavior, as interacting residues from PsaC are missing. A minimum of 20 C-terminal amino acids of PsaC had to be used in combination with subunits PsaA and PsaB to achieve proper binding in Alphafold predictions (Supplementary Fig. 5 b). While these *in silico* data might indicate weak binding strength of the PsaC-tag, the simulated results need to be viewed with care, as protein fold prediction is most reliable for native protein folding (such as PSI core subunits), but struggles with synthetic combinations of proteins (such as the hydrogenase fusion moieties). Hence the *in vivo* testing of the approach is still worthwhile.

Figure S6: Schematic overview of PSI cofactors and their electron transfer lifetimes. **a:** Schematic representation of PSI from *Synechocystis* sp. PCC 6803 with the relevant components for electron transfer from P_700_. Relative sizes are not to scale. **b:** Edge-to-edge distance, potentials and ranges of charge recombination times of PSI cofactors ^9,20,21^. Solid lines represent forward electron transfer lifetimes, while dashed lines represent the charge recombination time to P_700_^+^. Varying recombination times for the F_X_ in exposed (*) and regular (**) assembly state are displayed.

Table S1: Primer sequences used for cloning

| Primer name | Sequence | Fragment amplified | Purpose |
| --- | --- | --- | --- |
| psaC-upout1 | CTATAGGGCGAATTGGGTACCCACTGTAGAAGGCATAAACGAT | Upstream recombination site | Introduction of rhamnose-inducible promoter cassette into *psaC* locus |
| SpR-PsaC-up | TAGCGAGGGCGAGTGACTTTGAGCTTGTAACCAAGAAAAAC |  |  |
| psaC-up-SpR | AAAGTCACTCGCCCTCGCTAGATTTTAATGCG | SmR-cassette, RhaS-activator, rhaB-promoter |  |
| psaC-rhaB | TATGGGACATGTAACCTCCACTACTCTAGTATTCATTACGACC |  |  |
| rhaB-PsaC-down | AGGTTACCATATGTCCCATAGTGTAAAAATTTACGATACCTGTATTGGTTG | *psaC*, downstream recombination site |  |
| Psacdownout1 | AGGGAACAAAAGCTGGAGCTGATTATTTCGTG |  |  |
| PsaFout1neu | CTATAGGGCGAATTGGGTACGCAAAGCTTGGTCGAGACAGGTATTAATTAAAA | 5’ part of *psaF* (upstream recombination site) | Fusion of *hoxY* to *psaF* |
| G-rev HoxY-PsaF132 | CCGGAGCCACCGGAGCCACCCACCAGTTTGCCGGAGGTGTATT |  |  |
| 12 AA-G PsaF132-HoxY | GGTGGCTCCGGTGGCTCCGGTGGCTCCGGTGGCTCCGCTAAAATTCGTTTTGCTACCGTT | GGS linker [12 aa], *hoxY*, Chen-linker [5 aa] |  |
| C-rev PsaC-HoxY182 | GACACACTGCCGGATTCTTTACCAAATTTGATCATTGCTCGCCCTT |  |  |
| 5 AA-C HoxY182-PsaC | AAAGAATCCGGCAGTTTGGGTGCCGAAACCACCCGCAGTA | Chen-linker [5 aa], *psaC*-C-terminus, TST-hoxH, EmR-cassette, 3‘ part of *psaF* (downstream recombination site) |  |
| NaeI-PsaFout2 | CGAAGCAGGGTTATGCAGCGGAAAGTATACCTTAACCGCCAAAATCTAGGGGCCAGAATCGCCAA |  |  |
