# Supplementary material for: Removal of Photosystem I Subunit PsaE Leads to Photosynthetic Hydrogen Production in a Cyanobacterial PsaF‐HoxYH Fusion Strain
Source: Plant Cell Environ. 2026 Jun 9;49(9):6850–8. doi: 10.1111/pce.70659 (PMC13436535; doi:10.1111/pce.70659)
Supplement: Supplementary file 2 [file PCE-49-6850-s008.doc]

| Primer name | Sequence | Fragment amplified | Purpose |
| --- | --- | --- | --- |
| psaC-upout1 | CTATAGGGCGAATTGGGTACCCACTGTAGAAGGCATAAACGAT | Upstream recombination site | Introduction of rhamnose-inducible promoter cassette into *psaC* locus |
| SpR-PsaC-up | TAGCGAGGGCGAGTGACTTTGAGCTTGTAACCAAGAAAAAC |  |  |
| psaC-up-SpR | AAAGTCACTCGCCCTCGCTAGATTTTAATGCG | SmR-cassette, RhaS-activator, rhaB-promoter |  |
| psaC-rhaB | TATGGGACATGTAACCTCCACTACTCTAGTATTCATTACGACC |  |  |
| rhaB-PsaC-down | AGGTTACCATATGTCCCATAGTGTAAAAATTTACGATACCTGTATTGGTTG | *psaC*, downstream recombination site |  |
| Psacdownout1 | AGGGAACAAAAGCTGGAGCTGATTATTTCGTG |  |  |
| PsaFout1neu | CTATAGGGCGAATTGGGTACGCAAAGCTTGGTCGAGACAGGTATTAATTAAAA | 5’ part of *psaF* (upstream recombination site) | Fusion of *hoxY* to *psaF* |
| G-rev HoxY-PsaF132 | CCGGAGCCACCGGAGCCACCCACCAGTTTGCCGGAGGTGTATT |  |  |
| 12 AA-G PsaF132-HoxY | GGTGGCTCCGGTGGCTCCGGTGGCTCCGGTGGCTCCGCTAAAATTCGTTTTGCTACCGTT | GGS linker [12 aa], *hoxY*, Chen-linker [5 aa] |  |
| C-rev PsaC-HoxY182 | GACACACTGCCGGATTCTTTACCAAATTTGATCATTGCTCGCCCTT |  |  |
| 5 AA-C HoxY182-PsaC | AAAGAATCCGGCAGTTTGGGTGCCGAAACCACCCGCAGTA | Chen-linker [5 aa], *psaC*-C-terminus, TST-hoxH, EmR-cassette, 3‘ part of *psaF* (downstream recombination site) |  |
| NaeI-PsaFout2 | CGAAGCAGGGTTATGCAGCGGAAAGTATACCTTAACCGCCAAAATCTAGGGGCCAGAATCGCCAA |  |  |
